# Visual error augmentation enhances learning in three dimensions

**DOI:** 10.1186/1743-0003-8-52

**Published:** 2011-09-02

**Authors:** Ian Sharp, Felix Huang, James Patton

**Affiliations:** 1 Department of Bioengineering, University of Illinois at Chicago, 218 SEO, MC 063, 851 South Morgan Street, Chicago, Illinois 60607-7052, USA; 2 Rehabilitation Institute of Chicago (RIC), 345 East Superior, Rm. 1406, Chicago, IL 60611-2654, USA

## Abstract

Because recent preliminary evidence points to the use of Error augmentation (EA) for motor learning enhancements, we visually enhanced deviations from a straight line path while subjects practiced a sensorimotor reversal task, similar to laparoscopic surgery. Our study asked 10 healthy subjects in two groups to perform targeted reaching in a simulated virtual reality environment, where the transformation of the hand position matrix was a complete reversal--rotated 180 degrees about an arbitrary axis (hence 2 of the 3 coordinates are reversed). Our data showed that after 500 practice trials, error-augmented-trained subjects reached the desired targets more quickly and with lower error (differences of 0.4 seconds and 0.5 cm Maximum Perpendicular Trajectory deviation) when compared to the control group. Furthermore, the manner in which subjects practiced was influenced by the error augmentation, resulting in more continuous motions for this group and smaller errors. Even with the extreme sensory discordance of a reversal, these data further support that distorted reality can promote more complete adaptation/learning when compared to regular training. Lastly, upon removing the flip all subjects quickly returned to baseline rapidly within 6 trials.

## Background

Since the beginning of tool use, humans have been challenged with operating external devices that do not necessarily match natural limb movement. For example, through repetitive practice a novice computer user has to learn the remapping of anterior mouse motion to vertical cursor motion on the screen. Such repetitive experiences result in the learning of a neural representation that predicts the consequences of motor actions. Improving the efficiency of this learning process has been a remarkable area of research in neural engineering [1].

Recent studies have demonstrated error augmentation (EA) during repetitive practice can lead to faster and more complete learning for both visual [2] and haptic [3] augmentation. However, this research evaluated EA in small environmental distortions, typically a rotation of the visual field of 30 to 60 degrees. Yet distortions in everyday life commonly feature larger and often nonlinear distortions, or even complete reversals. For example, this is the case when surgeons perform laparoscopic surgery. Laparoscopic surgery requires the surgeon to learn that moving the handle of the instrument causes the tool tip to move in the opposite direction at a scaled distance and altered mechanical advantage, known as the fulcrum effect.

On the other hand, EA does not always cause more effective learning. Studies have shown that the process may not be effective for large errors [2]. Large errors are relevant in this study, because in a flip paradigm there are large errors initially, without the addition of EA. We wanted to know whether the presented feedback, under a paradigm where errors are large, would still continue to inform the remapping process. If not, remapping hence may be limited to the scale of the distortion [2]. It remains to be seen whether the augmentation learning process loses its effectiveness in tasks that involve large distortions. At the same time, the benefits of augmenting error may have the greatest impact on tasks that require large distortions, such as laparoscopy where large discrepancies in motor mappings occur.

In this study, we addressed larger distortions in which subjects learned a full reversal. We evaluated whether the learning process could be enhanced using error augmentation. The results of our study suggest that error augmentation assisted learning lead to improved performance by the end of training, even in large distortions.

## Methods

**Figure 1:**
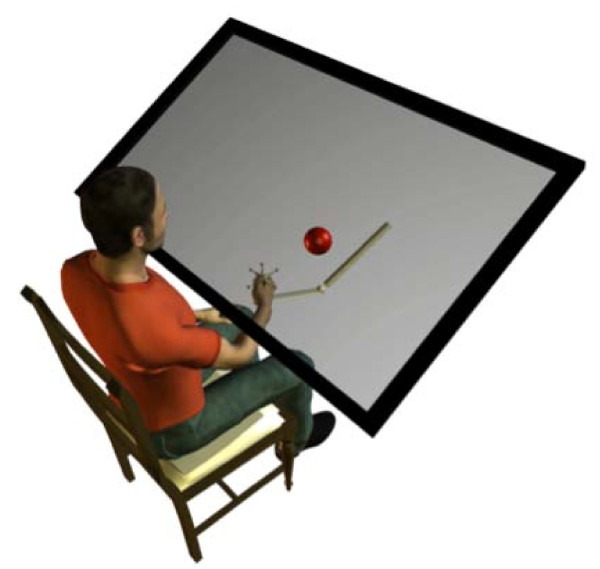
**VRROOM **.Virtual Reality and Robotic Optical Operations Machine.

This experiment utilized a three-dimensional, large-workspace haptics/graphics system called the Virtual Reality and Robotic Optical Operations Machine (VRROOM). VRROOM is an integrated system combining display environment, robotic forces, and tracking of limb movement (Figure 1). VRROOM's visual display system, the Personal Augmented Reality Immersive System (PARIS), was developed in the Electronic Visualization Lab at the University of Illinois at Chicago. PARIS is currently the highest quality system available, with a high-fidelity PHANToM 3.0 haptics device, Flock of Birds magnetic tracking devices, and its 2000-lumen cinema-quality digital projector that provides a 120-degree-wide field of view, described in more detail here [3].

Using this equipment we conducted a targeted reaching experiment on human subjects. Each subject signed a consent form that conformed to federal and University guidelines. We asked 10 healthy subjects with no history of orthopedic or neurological disorders to perform targeted reaching in a virtual reality environment, where the transformation of the hand position matrix was a complete reversal -- rotated 180 degrees about an arbitrary axis (hence 2 of the 3 coordinates were reversed). There were 10 subjects in each group. Each subject sat in front of the haptics/graphics system and performed a total of 620 targeted reaching trials, while holding the handle of the robot. There were a total of 5 targets located at the vertices of a tetrahedron, where only one target was made visible at a time. The distance between vertices was 0.15 m.

The experiment consisted of the following four phases in series: baseline, flip, evaluation, and washout each of which are described in detail below. Each phase consisted of a set of trials (discrete movements to a target), the first of which referred to the initial window, and the last of which are referred to as the end window. Both windows include 10 trials. Each trial began with the appearance of a target, and ended once the subject's cursor reached and resided within the current target for 0.5 seconds. There was no limit on the amount of time spent on completing a trial. The duration of the entire experiment was approximately one hour. During the first 60 trials (the baseline phase) subjects were allowed to familiarize themselves with the environment. No visual error augmentation was used, and the movement of the subjects hand to where the cursor appeared on the screen was a 1:1 gain for both groups.

During the next phase (the flip phase), the next 480 trials were performed where a full 180 degree rotation about an arbitrary z-axis took place. This means that when the subject moved their hand to the left, the cursor moved to the right; when they moved their hand to the right, the cursor moved left; when the subject moved their hand up, the cursor moved down; and when the subject moved their hand down, the cursor moved up. Movements of depth remained the same. During the flip phase, only the treatment group received error augmentation. The "error" that was augmented was the subjects' deviation from the "ideal point-to-point reaching trajectory". This ideal trajectory was assumed to be a straight line from target to target. The gain of the error augmentation was set to 2. Therefore, for every cm the subject deviated from the ideal straight line trajectory, the cursor on the screen deviated 2 cm. Lastly, all subjects were informed of both: the onset of the flip phase, and the transformation effect it would have.

During the next phase (the evaluation phase), 20 trials were performed within the flip phase paradigm. The treatment group had their error augmentation removed. It is important to note that the task was still a reversal during the evaluation phase. All end-performance comparisons after the 500 trials of training were analyzed in the evaluation phase. This is critical, as both the control and EA groups experienced the same flip paradigm with a gain of 1:1, therefore allowing us to properly compare performance.

During the last phase (the washout phase), the flip paradigm was removed and reaching returned to normal for the final 60 trials.

Different error metrics reveal how training alters different features of movements. For instance time per trial does not address the spatial accuracy of the movement or peak velocity. Spatial accuracy does not address the smoothness of the motion. Because we were interested in comparing the learning between groups we selected the simple metrics of: time per trial, maximum perpendicular distance, number of times the subjects stopped moving their arm per trial (NTSS), and finally initial direction error (IDE).

### Measures of error

We evaluated the *completion time *of each movement, along with maximum perpendicular distance from the straight line (*MxPd*) connecting the starting point and the target. We also looked at the number of times the subject stopped (*NTSS*), defined by the number of intervals where hand speed dropped below 0.06 meters/second. Finally, we evaluated the launch direction *initial error*, defined as the angle between the ideal straight line to the target and the vector formed from the starting point (defined by initial velocity going above .06 m/s) and a point 100 ms after that.

### Statistics

Error metrics were compared between groups by averaging performance during the last 10 trials of the evaluation phase for each subject. The mean of averages was then compared for each group. To determine if the group improved, the Mann-Whitney U test was performed on window size of 5 data points per subject. The alpha level to test for significance was set at 0.05. T-tests were not used, because both the Kolmogorov-Smirnov and Lilliefors test rejected the hypothesis that our data was normally distributed at the 5% significance level.

## Results

**Figure 2:**
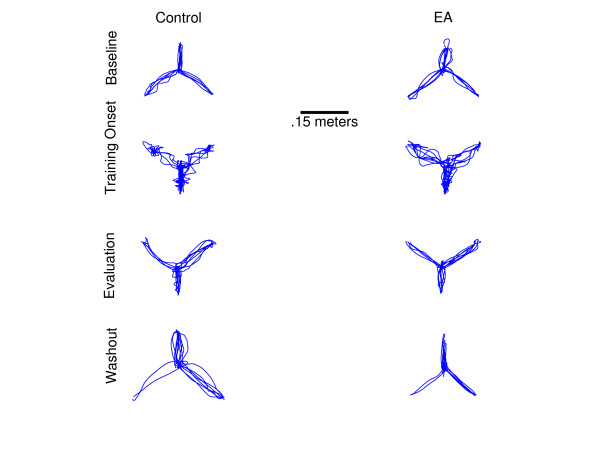
**Typical movement profiles**. Each plot above displays the expected movement profiles at the onset of a particular phase. The left column displays the control group, whereas the right column displays the EA group. Row one shows the baseline phase, the second row shows the onset of training, the third row shows the end of training, and the last row shows the washout phase. Note that during the training phase the EA group moves smoother than the control group.

As expected, groups performed well during baseline. No significant difference was achieved between groups for any error metric for the baseline phase's initial window, nor the baseline phase's end window (Figure 2).

**Figure 3:**
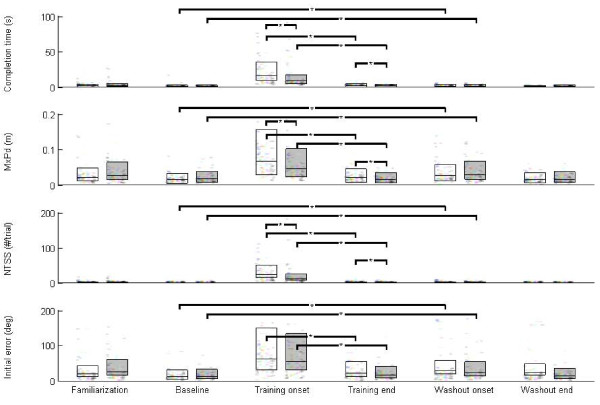
**Average subject errors across different phases of the experiment**. Error metrics decrease as training progresses. The horizontal line within each bar represents the average group performance over a 10 trial window. The top and bottom of bars represent the 25th and 75th percentiles. The control group is depicted in white, while the EA group is depicted in grey. Every coloured dot within the box represents a different subject. The first 10 trials for each subject are overlaid semitransparent. Horizontal lines and asterisks' are drawn to signify significance between and within phases.

The EA group performed trials quicker in the onset of training by 6.8 s (p = 6e-4), had a reduced maximum perpendicular distance (MxPd) by 2 cm (p = .002), and had fewer stops by 10 stops per trial (p = 0.003) (Figure 3). Initial direction error did not differ between groups in this phase (p = 0.4) (Figure 2).

In the evaluation phase's end window, the EA group performed trials quicker by 0.4 s (p = 0.0003), had a reduced maximum perpendicular distance by 0.5 cm (p = 0.0002), and had fewer stops by 0.6 (p = 0.005). Initial launch error did not achieve significance (p = 0.1) (Figure 2). These data suggest that the EA group was able to reach their end target quicker than the control group and was able to reach closer to a straight-line trajectory during this visual transformation; while stopping less frequently.

Both groups showed improvement from the initial training window to the end evaluation window for each error metric (Figure 3). The control group improved time per movement by 13 s (p = 5e-17), maximum perpendicular distance by 0.045 m (p = 1e-14), number of stops by 21 (p = 4e-16), and initial direction error by 40 degrees (p = 6e-9). Where the EA group improved time per movement by 7 s (p = 2e-17), maximum perpendicular distance by 0.03 m (p = 7e-14), number of stops by 11 (p = 1e-16), and initial direction error by 36 degrees (p = 4e-9) (Figure 2). Although the treatment group improvements were less over training, the treatment group initially started training with less error for most metrics: including movement time (p = 6e-4), maximum perpendicular distance (p = 0.002), and number of stops (p = 0.003). However, initial direction error did not differ between the groups when training began (p = 0.4). Large percent reductions in error occurred within the first 10 trials for the treatment group, where time per movement decreased 92%, maximum perpendicular distance decreased 76%, number of stops decreased 97% and initial direction error decreased 76%.

For all error metrics, after-effects washed out quickly -- below 2 standard deviations of the baseline mean within 6 trials for each subject. The first 2 trials of the washout phase were compared to the last 2 trials of baseline performance to determine after-effects. In spite of this, though, all subjects showed significant after-effects, with 0.8 seconds longer per movement (p = 0.0008), maximum perpendicular distance 2 cm larger (p = 0.002) than baseline, 1.4 more stops per trial (p = 0.01), and initial direction error averaged 28 degrees larger than baseline (p = 0.01). In the washout phase's initial window, between groups, no significant difference was achieved for any error metric (Figure 2).

We further inspected washout in 3 of the subjects by providing cues at the onset of the washout phase to observe performance. Each of these subjects moved their arm immediately toward the target, showing no significant after-effects (i.e., no significant differences in any of the measures from baseline).

## Discussion

Previous studies have already shown benefits of training with error augmentation, providing evidence that the motor system depends on error information to drive motor adaptation. Our findings, however, further these conclusions for the important special case of large sensorimotor discrepancy--in this case a complete movement reversal. Our results showed that all subjects improved during training in each of our metrics. However, groups exhibited important differences in both the initial training and evaluation phase. Our main finding was that the group treated with error augmentation exhibited superior performance in the evaluation phase that persisted even when their augmentation was removed.

Our analysis of training data revealed learning that clearly differed between groups. At initial exposure to the reversal task, the EA group stopped less frequently, and reached their targets more quickly. Other researchers have observed that subject will "stop-and-think" in the event of large movement errors, perhaps to evaluate recent movements and sub-movements and then re-plan movement strategies [4,5]. For our results, we speculate that because the EA group perceives their mistakes more clearly, they require less resetting and iterative attempts at performing straight line reaching movements (Figure 3).

Our use of several error metrics has revealed different aspects of learning. Practical measures of performance, time-per-movement, the number of times the subject stopped, and maximum perpendicular distance could include influences from both feedforward planning and online control. In contrast, the initial direction error focuses on the initial feedforward action, revealing planning differences between groups. Finally, the NTSS metric is unique in that it captures how the motor system copes with successive attempts at movement correction within a trial. This metric might reflect the degree to which subjects must reset plan new sub-movements. Taken together, our metrics suggest that EA contributes to both more accurate feedforward planning, and more robust online corrections.

Analysis of performance during training indicated primarily abrupt reductions in error, rather than gradual adaptation. While investigations of motor learning typically report error reduction that exhibit patterns of exponential decay, our data shows varying trends of error reduction across subjects. These data may indicate a different form of learning in this experiment. Early in training, subjects of the EA group exhibited a rapid improvement in performance, with further improvements occurring over the course of training. Other investigators have found cases in which learning could not be described in terms of exponential decay functions, where "no meaningful value for τ could be calculated" and "the problem could not be alleviated by using double exponentials [6]." However, others suggest two [7] (Smith and Shadmehr) or more [8] (Schweighofer) learning processes. Our data suggests immediate performance changes in the error-augmented group when compared to the control group. Furthermore, in terms of learning transfer, the EA group exhibited improved error metrics during the evaluation phase, despite having EA removed. The retention of performance gains provides support that error augmentation could have practical applications for rehabilitation and other forms of motor skill training.

The abrupt changes in error in washout are consistent with the hypothesis that there are two or more parallel learning processes involved in acquiring such skills in transformation tasks. The washout phase showed small but significant initial error for all metrics, which rapidly diminished for all subjects within 3 trials. Once training ended, there was a mild difference in the end of baseline performance when compared to the onset of the washout phase for most error metrics. Rather than a pure cognitive switch, these data imply that multiple competing models may simultaneously be represented. Researchers such as Wolpert and Kawato [9] have hypothesized multiple paired forward and inverse models in human motor control. While a gradual de-adaptation after-effect has been claimed as supporting evidence that "error dependent learning" has taken place in other visual feedback error studies [10], we did not observe this in the present experiment. Others have found that contextual interference (CI) is enough to change internal models "due to [their] improved capacity to actively prepare motor responses" [11]. While still others have found evidence that suggests the nervous system estimates the relevance of information using Bayesian statistics [12]. Therefore, it could be that this type of learning represents a mode separate from control models that involve incremental adjustments of control parameters, allowing for more rapid switch back to the normal world.

The findings of this study could have broad implications for training in applications ranging from surgical training to sports, teleoperation, and rehabilitation, where large sensorimotor discrepancies must be learned. Error augmentation experiments may also be an excellent method for rehabilitation training. As this study suggests, such internal model modulation depends on understanding a number of unexplored factors, such as rates of learning in the pathological state. Optimal distorted reality treatment parameters are not yet known, and leave opportunities for research in wider applications in areas such as sports, teleoperation, rehabilitation, piloting and surgical training. It remains to be seen whether error augmentation using forces might have a similar beneficial effect. What is clear in the present study is that visual error augmentation approaches are viable even in the face of the large sensory discrepancies such as the reversal experienced in this study.

## Competing interests

The authors declare that they have no competing interests.

## Authors' contributions

ICS tested the subjects, analyzed the subjects, analyzed the subjects' data and led the writing of this paper. JLP provided the funding, was responsible for receiving human subjects' approval, guided analysis, and assisted in writing of this paper. FCH assisted in the data analysis and editing. All authors read and approved the final manuscript
